# Corticotropin Releasing Factor Type 1 and 2 Receptor Signaling in the Medial Prefrontal Cortex Modulates Binge-Like Ethanol Consumption in C57BL/6J Mice

**DOI:** 10.3390/brainsci9070171

**Published:** 2019-07-19

**Authors:** Stacey L. Robinson, Carlos A. Perez-Heydrich, Todd E. Thiele

**Affiliations:** 1 Department of Psychology & Neuroscience, The University of North Carolina, Chapel Hill, NC 27599, USA; 2 Bowles Center for Alcohol Studies, The University of North Carolina, Chapel Hill, NC 27599, USA

**Keywords:** binge drinking, corticotropin releasing factor, corticotropin releasing hormone, mice, ethanol, alcohol, drinking in the dark, CRF1R, CRF2R, medial prefrontal cortex

## Abstract

Corticotropin releasing factor (CRF) signaling via limbic CRF1 and 2 receptors (CRF1R and CRF2R, respectively) is known to modulate binge-like ethanol consumption in rodents. Though CRF signaling in the medial prefrontal cortex (mPFC) has been shown to modulate anxiety-like behavior and ethanol seeking, its role in binge ethanol intake is unknown. Here, we used “drinking-in-the-dark” (DID) procedures in male and female C57BL/6J mice to address this gap in the literature. First, the role of CRF1R and CRF2R signaling in the mPFC on ethanol consumption was evaluated through site-directed pharmacology. Next, we evaluated if CRF1R antagonist reduction of binge-intake was modulated in part through CRF2R activation by co-administration of a CRF1R and CRF2R antagonist. Intra-mPFC inhibition of CRF1R and activation of CRF2R resulted in decreased binge-like ethanol intake. Further, the inhibitory effect of the CRF1R antagonist was attenuated by co-administration of a CRF2R antagonist. We provide novel evidence that (1) inhibition of CRF1R or activation of CRF2R in the mPFC reduces binge-like ethanol intake; and (2) the effect of CRF1R antagonism may be mediated via enhanced CRF2R activation. These observations provide the first direct behavioral pharmacological evidence that CRF receptor activity in the mPFC modulates binge-like ethanol consumption.

## 1. Introduction

Binge drinking is defined by the National Institute on Alcohol Abuse and Alcoholism (NIAAA) as a pattern of consumption that produces blood ethanol concentrations (BECs) greater than 0.08% (80 mg/dL) within an approximately two-hour period. Excessive binge drinking is of concern in the military [1], postgraduate community [2], and across the spectrum of socioeconomic populations [3]. Of further importance, binge ethanol consumption is associated with a variety of health consequences and is widely recognized as a significant risk factor for the development of ethanol dependence [4,5,6]. Given this, assessment of neural targets for pharmacological treatments to help reduce binge ethanol consumption is a critical area of ongoing research. In rodents, the neural mechanisms underlying binge ethanol consumption can be studied through the “drinking in the dark” (DID) model. While non-ethanol dependent rodents typically avoid engaging in excessive ethanol intake, in the DID model, starting at 3 h into their dark cycle, C57BL/6J mice are given access to a 20% ethanol solution for 2 to 4 h (for review see [7]). During this time, these mice will reliably consume levels of ethanol sufficient to produce BECs of >100mg/dl and will exhibit behavioral signs of intoxication [8].

The corticotropin-releasing factor (CRF) system is a target for pharmacological treatment of binge drinking behavior that has received considerable attention in the recent past [9]. CRF is a 41-amino acid poly-peptide widely expressed throughout the brain and is best known for its role as a pro-stress neuropeptide [10]. CRF is thought to signal predominantly through the CRF 1 receptor (CRF1R) and CRF receptor 2 (CRF2R) via G_s_-protein, though the receptors are capable of acting through a variety of G-proteins in a potentially region-specific manner [11,12]. This system has been shown to modulate binge-like alcohol consumption in non-dependent animals in a region-specific manner [13,14,15,16], as well as to play a critical role in the development of ethanol dependence and during abstinence-induced withdrawal from chronic ethanol use [9,17]. The CRF system is thus poised to significantly contribute to each stage of alcohol use disorder (AUD), from initiation to dependence. However, the specific neural circuits through which CRF modulates binge-like ethanol consumption remains an area of intense investigation.

Given the well-known pro-stress role of CRF, a primary focus of the role of CRF in alcohol related behaviors have been signaling within the hypothalamus and limbic regions associated with emotional regulation [18,19]. Notably, recent evidence has shown CRF signaling in cortical regions responsible for executive function and decision making is also significantly engaged during binge drinking behavior [20]. The CRF system is abundantly expressed within the medial prefrontal cortex (mPFC), a brain region which has been evolutionarily conserved between rodents and humans [21] to serve a critical role in the regulation of goal-directed behavior and inhibition of inappropriate actions [22,23]. Structural and functional alterations are observed in the mPFC in alcohol dependent individuals, lending further support for a role of this region in alcohol abuse behaviors [22,24]. A subpopulation of mPFC gamma-Aminobutyric acid (GABA)-ergic neurons produce CRF and synapse onto both excitatory projection neurons and other interneuron populations [11,25,26]. Notably, CRF1R and CRF2R activation may differentially alter activity of distinct cell populations, potentially contributing to the opposing roles these two receptors play in a number of behaviors including consumption and anxiety [27]. For instance, within the dorsal raphe nucleus (DRN), CRF1R activation alters GABAerigc while CRF2R activation alters serotonergic signaling [28,29]. Of interest to this work, CRF2R mRNA has been detected only at low levels within the mPFC [11]. In contrast, CRF1R is richly expressed in the region and, further, CRF displays eight-fold higher affinity for CRF1R relative to CRF2R [30]. However, recent work has demonstrated mPFC CRF2R activity modulates cocaine reinstatement and CRF2R, but not CRF1R, mRNA is significantly increased in the mPFC following sub-chronic nicotine exposure [31,32]. As these works suggest a role for mPFC CRF2R in drug abuse behavior despite low expression, we sought to examine the role of both CRF1 and CRF2 receptor types.

The role of mPFC CRF in regulating alcohol-seeking in dependent animals has been demonstrated [22], however, a role in binge-like ethanol intake in non-dependent animals has yet to be evaluated. As previous work in our laboratory has demonstrated an important role of the anti-stress neuropeptide, neuropeptide Y (NPY) in the mPFC in binge-like ethanol intake, we hypothesized altering CRF receptor activity in the mPFC through antagonism of CRF1R or activation of CRF2R would impair binge-like consumption of ethanol [33].

## 2. Materials and Methods

### 2.1. Animals

Male and female C57BL/6J mice (Jackson Laboratories (Bar Harbor, ME)) housed individually in an AAALAC accredited vivarium (22 °C; reversed 12:12h light:dark cycle, lights on: 20:00) with ad libitum access to Prolab^®^ RMH 3000 (Purina LabDiet^®^; St. Louis, MO, USA) and water (unless otherwise stated) were used in all experiments. On arrival, animals were allowed to acclimate to the environment for ≥1 week. Separate cohorts of animals were used in each experiment. The University of North Carolina Institutional Animal Care and Use Committee approved of and we followed the Guidelines for the Care and Use of Laboratory Animals in all procedures.

### 2.2. “Drinking in the Dark” Procedures

A 4-day DID paradigm was used to model binge intake as previously described [34]. Three hours into the dark cycle (11:00 AM), water bottles were removed sipper tubes containing 20% (*v*/*v*) ethanol or 3% (*w*/*v*) sucrose in tap water were place in each animal homecage for 4 h or 2 h. On the fourth day of each cycle (test day), animals were treated with drug or appropriate vehicle ~30 min prior to bottles on. Each 4-day DID cycle was separated by three days. Tail blood samples (≈60 µL) were taken immediately following each ethanol test day to assess blood ethanol concentrations (BECs) (AM1 Alcohol Analyzer (Analox, London, UK)).

### 2.3. Open Field Procedures

**Figure 1:**
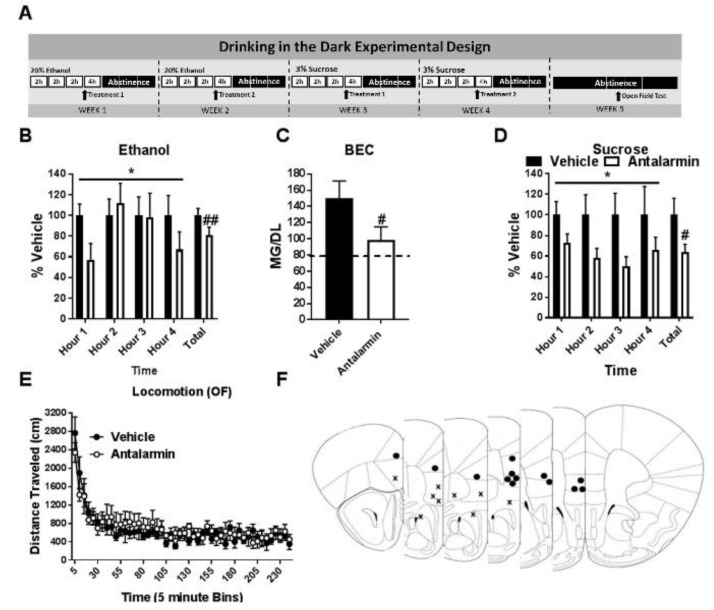
Corticotropin releasing factor receptor 1 (CRF1R) antagonism in the medial prefrontal cortex (mPFC) blunts binge intake without altering general locomotion. (**A**) Experimental timeline. (**B**) Antalarmin microinjection into the mPFC significantly reduced ethanol intake and (**C**) final blood ethanol concentration compared to vehicle. (**D**) Antalarmin further reduced sucrose intake across time compared to vehicle. (**E**) Antalarmin did not alter general locomotion (each symbol = 5 min bin, x axis labeled every 25 min). (**F**) Cannula placement checks (• = hit *N* = 13, X = miss *N* = 9). * = ANOVA treatment factor *p* < 0.05; # = *t*-test *p* < 0.05.

Behavioral testing occurred at least one week following DID sucrose testing (at least 3 weeks after final binge-ethanol exposure) (Figure 1A). Animals were assigned into groups that received the vehicle or drug treatment opposite to that received in the final sucrose consumption week and administered treatment identically as described for DID testing, save animals only underwent one open field test (no Latin-square design was used as described below). Test were performed 4–6 h after the initiation of their dark period. Animal movement in the open field was tracked using the VersaMax^®^ software program (AccuScan Instruments, Inc., Columbus, OH, USA) (measurements: 16.5 × 16.5 × 12 inches) over 2 h period with five-minute bin outputs. Total distance traveled was measured to assess the effect of treatment on locomotor activity.

### 2.4. Surgeries & Drug Administration

Surgeries were performed following standard Thiele lab procedures [33]. In short, animals were given intraperitoneal (i.p.) injections (1.5 mL/kg) of an anesthetizing cocktail of xylazine (10 mg/kg) and ketamine (100 mg/kg). In cannulation studies, using an Angle II™ Stereotax (Leica Instruments, Buffalo Grove, IL, USA), bilateral guide cannula (33 GA double cannula cut 4 mm below pedestal; dummy cannula: 4 mm with 0.5 mm projection; injector: 4 mm with 2 mm projection) (Plastics One; Roanoke, VA, USA) were lowered into the mPFC (AP: 1.7, ML: ±0.4, DV: −2.6). Mice recovered for ≥1 week before the start of the experiment.

Animals were allowed one DID cycle without drug administration to acclimate to the DID procedure. Starting the second week, on DID test days animals underwent microinjection of the experiment-specific drug or appropriate vehicle ~30 min prior to test start. Drugs were as follows: CRF1R antagonist antalarmin (Sigma-Aldrich, St. Louis, MO, USA) made up in 5% DMSO in sterile saline injected as 1 µg/0.5 µL/side; CRF2R agonist Ucn3 (Phoenix Pharmaceuticals Inc., Burlingame, CA, USA) in sterile saline injected as 60 pmol/0.4 µL/side; CRF1R antagonist NBI 35965 (Tocris, Minneapolis, MN, USA) in sterile saline injected as 30 pmol/0.3 µL/side; CRF2R antagonist K 41498 (Abcam, Cambridge, MA, USA) made up in sterile saline injected as 50 pmol/0.3 µL/side. To limit the number of microinjections animals underwent, single drug doses were chosen based on previous dose-response curve experiments performed in our lab ([35] or preliminary results). Experiments 1 and 2 used a 2 × 2 Latin-square design as previously described [33]. In brief, on test day 1 animals were randomly assigned to drug or vehicle treatment. On the following test day 2 of a second DID cycle animals then received the alternative treatment to allow for each animal to serve as its own control (vehicle vs drug treatment day) (See Figure 1A). Previous work in our lab has demonstrated stable DID ethanol intake for up to six weeks, allowing for analysis of treatment-dependent changes in intake across time [36]. Drug microinjections were performed with a Hamilton syringe (Hamilton, Reno, NV, USA) attached to a Harvard Apparatus PHD 2000 infusion pump (Harvard Apparatus, Holliston, MS, USA) and occurred at an infusion rate of 0.5 μL/min with injectors remaining in place for an additional 0.5–1 min to allow diffusion. Potential carry-over effects 24 h following injection were not specifically evaluated as animals entered a 3-day abstinence period following each test day. For cannula placement checks, brains were extracted by each mouse being administered 0.1 mL intraperitoneal (i.p.) injection ketamine/xylazine (6.67 mg/0.1 mL; 0.67 mg/0.1 mL; in 0.9% saline) and perfused transcardially using 0.1 M phosphate buffer saline (PBS; pH = 7.4) and 4% paraformaldehyde in PBS (pH = 7.4). After extraction, brains were post-fixed in 4% paraformaldehyde for 24–48 h then sectioned at 40 µm thickness (Leica VT1000S vibratome; Wetzlar, Germany). Cannula placements were verified by imaging of mounted sections with a digital camera (Roper Scientific), mounted on an optical microscope (Leica DM6000). Cannula location was defined as the end of the guide cannula plus 2 mm to account for injector projection length.

### 2.5. Statistical Analysis

GraphPad Prism (GraphPad Software, Inc. La Jolla, CA, USA) was used to analyze and graph all data, except three-way ANOVAs which were performed in SPSS statistics (IBM Analytics, Armonk, NY, USA). In experiment one and two two-way ANOVAs and Bonferroni post-hoc tests were used to evaluate treatment versus time during individual DID drinking hours, while T-tests were used to evaluate treatment effect on total intake and BECs. One-way ANOVA and Dunnett’s posthoc test were used in Experiment 3. All data are reported as the mean ± standard error of the mean and considered significant if *p* < 0.05 (two-tailed). Animals were removed from analysis if they were found to be a significant outlier as detected by a Grubbs test (Alpha = 0.05) or if cannula misplacement (uni or bilateral) was found. In cases where cannula became blocked between ethanol and sucrose studies animals were immediately perfused and ethanol results alone reported. Three-way ANOVA analysis found no significant effect of treatment order nor sex in any Latin-square design experiment, therefore these data were collapsed and analyzed by two-way ANOVA.

## 3. Results

### 3.1. Pharmacological Inhibition of CRF1R Selectively Reduces Binge-Like Ethanol Consumption

Two-way (treatment × time) ANOVAs were used to analyze the Latin Square data and paired *t*-test to analyze percent vehicle intake and BEC. Treatment timeline detailed in Figure 1A. mPFC microinjection of the selective CRF1R antagonist antalarmin reduced hourly and total (four hours combined) binge-like ethanol intake in (*N* = 13) (Treatment: F (1, 12) = 6.819, *p* < 0.05; Time: F (3, 36) = 0.8175, *p* > 0.05; interaction: F (3, 36) = 1.378, *p* > 0.05; Total *t*-test: *t* = 3.437 df = 12, *p* < 0.01) (average intake: vehicle: 5.9 ± 0.4 g/kg; treatment: 4.8 ± 0.5 g/kg) (Figure 1B). This reduction was sufficient to result in a similar significant decrease in BEC at the end of the test period (*t* = 2.429 df = 12, *p* < 0.05) (Figure 1C). Between ethanol and sucrose exposure one male subject developed a blocked cannula and was removed from the study. Decreased intake was likewise observed during hourly and total (four hours combined) sucrose consumption (*N* = 12) (Treatment: F (1, 11) = 5.029, *p* < 0.05; Time: F (3, 33) = 0.511, *p* > 0.05; interaction: F (3, 33) = 0.2539, *p* > 0.05; Total *t*-test: *t* = 2.236 df = 11, *p* < 0.05) (average intake: vehicle: 214.6 ± 34.7 mL/kg; treatment: 136.4 ± 16.9 mL/kg) (Figure 1D). Importantly, antalarmin did not alter general locomotion (Vehicle *N* = 7, Drug *N* = 5; Treatment: F (1, 10) = 0.1026, *p* > 0.05; Time: F (47, 470) = 15.39, *p* < 0.05; interaction: F (47, 470) = 0.8874, *p* > 0.05) (Figure 1E), suggesting drug effect was due to a selective reduction in consumption rather than mobility impairment. Impact of treatment on ethanol and sucrose intake by sex presented in (supplementary data Figure S2).

### 3.2. Pharmacological Activation of CRF2R Selectively Reduces Binge-Like Ethanol Consumption

**Figure 2:**
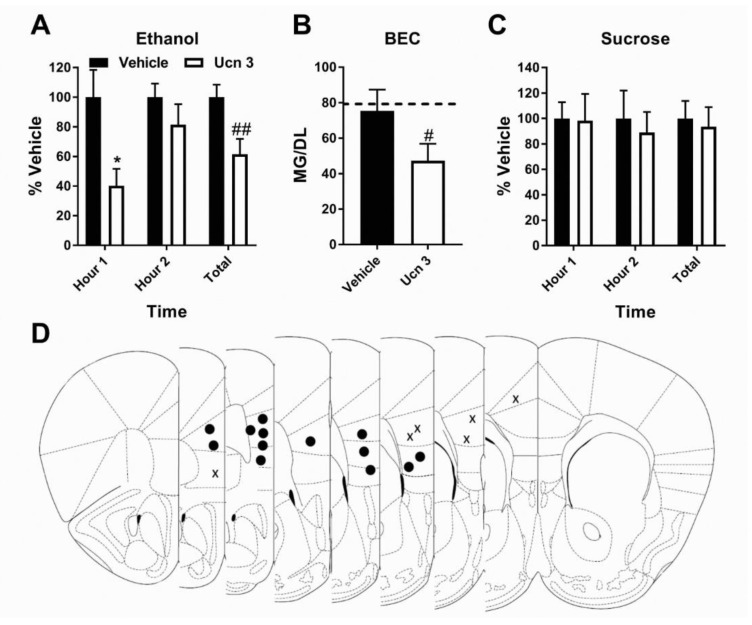
CRF2R agonism in the mPFC specifically blunts binge ethanol intake. (**A**) Urocortin 3 (Ucn3) microinjection into the mPFC significantly reduced ethanol intake and (**B**) final blood ethanol concentration compared to vehicle. (**C**) Ucn3 did alter sucrose intake. (**D**) Cannula placement checks (• = hit *N* = 13, X = miss *N* = 6). * = preplanned Bonferroni posthoc test *p* < 0.05; # = *t*-test *p* < 0.05; ## = *t*-test *p* < 0.01.

Two-way (treatment × time) ANOVAs were used to analyze the Latin Square data and paired *t*-test to analyze percent vehicle intake and BEC. As per the treatment timeline detailed in Figure 1A, save animals did not undergo locomotor testing. As the greatest reduction in consumption occurred in the first hour of Antalarmin treatment, DID testing was reduced to a 2 h window to better ensure capturing BECs related to when treatment was on board. We first confirmed the presence of CRF2R in the mPFC prior to experiment start (see supplementary data Figure S1). mPFC microinjection of the selective CRF2R agonist Ucn3 reduced total (2 h combined) binge-like ethanol intake in mice (*N* = 13) (Treatment: F (1, 12) = 11.8, *p* < 0.01; Time: F (1, 12) = 2.427, *p* > 0.05; interaction: F (1, 12) = 2.003, *p* > 0.05; Total *t*-test: *t* = 3.333 df = 12, *p* < 0.01) (average intake: v ehicle: 2.7 ± 0.2 g/kg; treatment: 1.7 ± 0.3 g/kg) (Figure 2A). Though the interaction did not reach statistical significance, a preplanned Bonferroni post-hoc test found this effect to be primarily driven by a reduction in ethanol consumption during the first hour of testing (*p* < 0.05). A similar significant reduction in BEC was observed (*N* = 13) (*t* = 3.333, df = 12, *p* < 0.01) (Figure 2B). Between ethanol and sucrose exposure, four male and one female subjects had blocked/dislodged cannula and were removed from study. In contrast, no impact of treatment was detected on sucrose consumption across individual hours or in total intake (2 h combined) (*N* = 9) (Treatment: F (1, 8) = 0.2332, *p* > 0.05; Time: F (1, 8) = 0.1186, *p* > 0.05; Treatment: F (1, 8) = 0.06814, *p* > 0.05; Total *t*-test: *t* = 0.4871 df = 8, *p* > 0.05) (average intake: vehicle: 98.1 ± 13.6 mL/kg; treatment: 91.8 ± 15.0 mL/kg) (Figure 2C). Impact of treatment on ethanol and sucrose intake by sex presented in (supplementary data Figure S3).

### 3.3. Co-Antagonism of mPFC CRF2R Attenuates mPFC CRF1R Antagonist Induced Reduction of Binge-Like Ethanol Consumption

**Figure 3:**
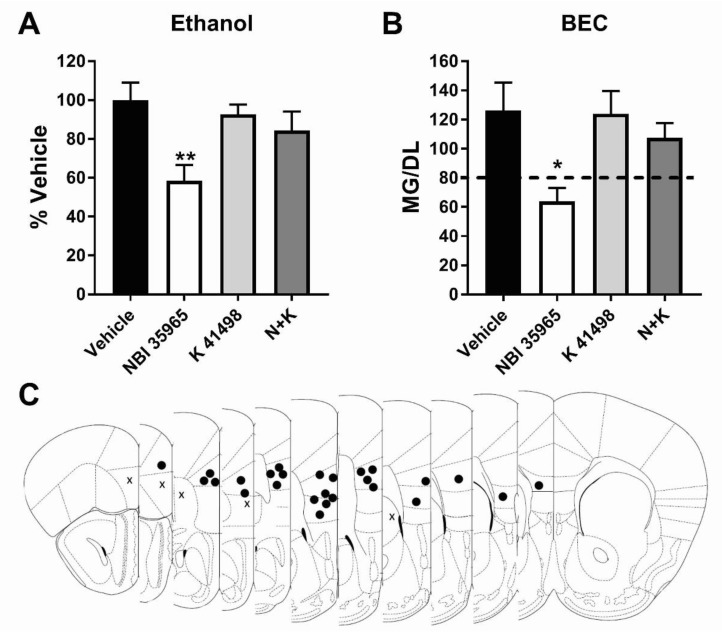
Co-administration of a CRF2R antagonist attenuates CRF1R antagonist blunting of binge ethanol intake. (**A**) A CRF1R antagonist alone, but not a CRF2R antagonist or CRF1R+CRF2R antagonist, reduced binge ethanol consumption and (**B**) final blood ethanol concentration compared to vehicle. (**C**) Cannula placement checks (• = hit *N* = 26, X = miss *N* = 5). * = One-way ANOVA Dunnett’s posthoc test *p* < 0.05; ** = One-way ANOVA Dunnett’s posthoc test *p* < 0.01.

Similar to Experiment 2, a reduced 2hr test day was used in this experiment. One-way ANOVAs with Dunnett’s post hoc test were used to analyze percent vehicle intake and BEC. Each subject underwent one DID cycle in this experiment, receiving one drug treatment. A CRF1R antagonist and CRF2R antagonist were administered alone or in combination to evaluate the role of CRF-interaction with CRF2R in the effects of CRF1R blockade on ethanol consumption. As previously performed within our lab, the CRF1R antagonist NBI 35965 was used in place of antalarmin to enable the use of an identical vehicle solution for the CRF1R and CRF2R antagonists [16]. Total ethanol consumption (2 h intake combined) was significantly affected by drug treatment (F (3, 22) = 4.664, *p* < 0.05). Post hoc found that, in keeping with results in Figure 1, mPFC microinjection of a CRF1R antagonist selectively reduced total binge-like ethanol intake compared to vehicle injection ((Vehicle *N* = 6; NBI 35965 *N* = 6) Vehicle vs. NBI 35965: *p* < 0.01). This reduction was attenuated by co-administration of a selective CRF2R antagonist ((NBI 35965 + K 41498 *N* = 7) Vehicle vs. N + K *p* > 0.05). In comparison, CRF2R antagonist administration alone did not impact ethanol consumption compared to vehicle ((K 41498 *N* = 7) Vehicle vs. K 41498 *p* > 0.05) (Total intake: Vehicle: 3.7 ± 0.3 g/kg; NBI 35965: 2.1 ± 0.3 g/kg; K 41498: 3.4 ± 0.2 g/kg; NBI 35965 + K 41498: 3.1 ± 0.4 g/kg) (Figure 3A). A similar effect was detected in BEC analysis ((Vehicle *N* = 6; NBI 35965 *N* = 6; K 41498 *N* = 7; NBI 35965 + K 41498 *N* = 7) F (3, 22) = 3.952, *p* < 0.05; Dunnett post hoc: Vehicle vs. NBI 35965 *p* < 0.05; Vehicle vs. K 41498 *p* > 0.05; Vehicle vs. NBI 35965 + K 41498 *p* > 0.05) (Figure 3B). Given the lack of impact of CRF2R agonism on sucrose consumption (Figure 2C), sucrose was not evaluated in this experiment. The impact of treatment on ethanol and sucrose intake by sex is presented in the (supplementary data Figure S4).

## 4. Discussion

This study was performed to assess the role of two CRFRs expressed in the mPFC and extensively demonstrated to regulate behaviors related to drug dependence in binge-ethanol intake. We found CRF1R antagonism in the mPFC non-specifically reduced intake of rewarding substances, decreasing both 20% ethanol and 3% sucrose consumption in male and female mice. In contrast, a CRF2R agonist in the mPFC specifically reduced ethanol, but not sucrose, intake. Further, co-administration of a CRF1R and CRF2R antagonist attenuated CRF1R antagonist-induced reduction of ethanol intake. This finding suggests the effect of intra-mPFC CRF1R antagonism may be predominantly mediated by redirecting CRF binding to CRF2R, an observation that we have previously described with respect to CRF receptor signaling in the ventral tegmental area (VTA) [16]. Results from these experiments suggest CRFR signaling in the mPFC parallel results observed in limbic regions critical to regulating ethanol-directed behaviors, in which both CRF1R and CRF2R activity actively modulate binge-intake [37,38]. While the mPFC is a well-recognized player in AUDs and other drug use disorders, it is traditionally associated with regulating drug seeking and later stages of dependence, such as relapse following withdrawal [23,39,40,41]. These experiments support the hypothesis that neuropeptides in this cortical region are actively engaged in modulating drug intake at a relatively early phase, prior to the development of ethanol dependence. Further, while previous work has demonstrated extensive dysregulation of the mPFC CRF system following sub-chronic nicotine or binge-ethanol exposure through use of mRNA analysis, this work is the first, to our knowledge, to use behavioral pharmacology to demonstrate the role of mPFC CRFRs in modulating binge ethanol intake [20,31].

Ethanol-induced alterations in anxiety and stress-related behaviors are hypothesized to underlie both high levels of binge intake and the eventual transition from casual ethanol use to dependence [9,42,43]. While the CRF system within limbic regions has been extensively demonstrated to play a critical role in mediating these underlying behaviors, mPFC CRF has recently emerged as an additional key player in regulating negative affect. Rodents will develop conditioned place aversion to a chamber paired with intra-mPFC infusion of CRF while CRF1R antagonist infusion will reverse avoidance of a stress-paired context [44]. CRF1R in the mPFC has also been shown to significantly contribute to stress-induced executive dysfunction [45], further supporting a role of the mPFC CRF system in modulating stress behavior such as that observed during withdrawal from chronic ethanol consumption. Important to the present work, mPFC CRF is further known to modulate cognitive function independent of stress as well as anxiety-like behavior in rodents [27,46]. Intra-mPFC infusions of CRF increased while infusion of a CRF1R antagonist attenuated anxiety-like behavior in male mice [29,47]. The relationship between CRF and anxiety may be dose dependent, as while lower doses of intra-mPFC CRF are anxiogenic, higher doses were found to be anxiolytic [29]. While this effect requires further study, the authors suggest it may be a result of CRF increasingly binding to the lower affinity CRF2R as dose increases, resulting in net anxiolysis [29]. Interestingly, in the above studies blockade of protein kinase A prevented the effects of intra-mPFC CRF infusion on both stress-induced executive dysfunction and angiogenesis, suggesting a potential common signaling pathway for CRF modulation of these behaviors [45,47].

In contextualizing our present work, it is important to note that while baseline CRF2R mRNA is only detected at low levels in the mPFC CRF1R, chronic or sub-chronic drug administration have been shown to significantly increase mPFC CRF2R mRNA [11,31,32]. While we did not directly assess alterations in CRF receptor expression here, these past works suggest the DID exposure our animals underwent prior to test day may have increased CRF2R expression, thus increasing the ability of CRF2R to modulate behavior. Quantitative analysis of mPFC CRF2R mRNA and protein concentration following ethanol exposure relative to baseline is therefore an important future experiment. Further, the specific expression pattern of CRF1R and CRF2R prior to and following ethanol exposure is another critical experiment. This is due to the evidence that CRF signaling via CRF1R and CRF2R likely modulates behavior by altering the release of distinct neurotransmitters/modulators, as observed in the DRN, or through changing excitability of mPFC cells projecting to distinct downstream targets, such as the extended amygdala (EA) or paraventricular nucleus of the hypothalamus [28,48,49,50]. Given CRF1R is at least eight-fold more sensitive to CRF relative to CRF2R [30], potential differences in receptor expression pattern would result in differing levels of ethanol-stimulated mPFC CRF release (i.e., low CRF release preferentially stimulating CRF1R and high CRF release also activating CRF2R) initiating significantly different alterations in mPFC downstream signaling.

CRF receptors have emerged as a critical pharmacotherapeutic target for the development of pharmacotherapies for alcohol use disorders. In brief, systemic administration of CRF1R antagonists reduces ethanol consumption in dependent and non-dependent animals, and further attenuates stress-induced relapse to ethanol seeking [9]. CRF2R agonists have been less commonly evaluated, but intracerebroventricular (ICV) administration has been shown to reduce binge intake in non-dependent and ethanol self-administration in dependent animals [13,14,51]. Subcortical regions such as the extended amygdala (EA) or hypothalamus have served as the primary focus of attention in understanding the role of CRFRs in binge-ethanol intake due to the important roles of these regions in modulating stress, emotionality, and other behaviors known to significantly influence ethanol intake prior to and during dependence [52]. Any pharmacotherapy targeting CRF signaling will presumably impact this system throughout the brain, highlighting the need to understand the role of CRF outside of the EA in alcohol directed behaviors. The mPFC is of particular interest in regulating binge ethanol intake as CRF binding protein, an important regulator of CRF signaling, is known to be significantly altered following binge exposure [20]. Notably, this present works suggests mPFC CRF receptor modulation of binge-ethanol intake functions similar to that previously observed by our lab in EA projections to the VTA [16]. Specifically, we previously demonstrated inhibition of binge-ethanol intake by a CRF1R antagonist into the VTA was attenuated by co-administration of a CRF2R antagonist [16]. We hypothesize this mechanism to be mediated by CRF1R antagonism redirecting CRF to bind at the lower affinity CRF2R. This apparent synergy between the role of CRFR in cortical and subcortical regions adds substantial support for targeting of the CRF system in alcohol use disorders.

A notable divergence between this previous work in the VTA and present findings is the source of CRF. It was found that chemogenetic inhibition of local CRF interneurons within the VTA did not alter ethanol intake, but rather this effect was mediated by CRF arriving from EA afferent projections [16]. While inhibition of mPFC CRF release was not directly assessed in this current work, we speculate based on previous literature suggesting local GABAergic interneurons serve as the primary source of mPFC CRF that, in contrast to the VTA, these neurons serve as the source of CRF receptor activation in the mPFC [25]. Previous studies have suggested that alternative sources of mPFC CRF may arise from the brainstem, such as from the CRF-expressing laterodorsal tegmental nucleus of the pons [53]. Recent work has further demonstrated a CRF-positive projection from the mPFC to the nucleus accumbens, suggesting a potential novel source of mPFC CRF distinct from the local interneuron population [54]. Further investigation is needed to determine the precise source of CRF relevant to these present findings. However, given there is at present little evidence for a CRF-expressing EA→mPFC projection [18,55], these results suggest that while CRF throughout the reward system plays an important role in binge-like ethanol intake the CRF pool mediating these effects in cortical and limbic regions are likely distinct.

The majority of previous work on CRF modulation of ethanol intake has been performed exclusively in male subjects. This focus on male rodents is notable as work in the past decade has found sex-specific differences in CRF system expression and activity [56]. For instance, relative to males CRF1R in females has been shown to be most sensitive to lower concentrations of CRF and more resistant to internalization following exposure to high CRF concentrations at baseline [57]. While no sex differences were observed in this present work, the relatively low N of each sex is a caveat which must be considered in the interpretation of this work. Notably, a trend towards a significant impact of sex as a factor (*p* = 0.07) was detected in ethanol intake in experiments 1 and 2 (Supplemental Figures S2 and S3). CRF1R antagonist administration in the first hour was found by Bonferroni’s multiple comparisons test to significantly reduce ethanol intake only in females, consistent with the higher baseline sensitivity of female CRF1R (Supplementary Figure S2). Further, following CRF2R agonist administration, Bonferroni’s multiple comparisons test performed on total ethanol intake (Supplementary Figure S3), detected a significant reduction in male, but not female, intake. This is in contrast to the first hour of intake, in which agonist treatment significantly reduced ethanol consumption equally in males and females. This may potentially suggest a difference in CRF2R sensitivity/internalization or drug metabolism between females and males. Alternatively, this may suggest the higher sensitivity of CRF1R in females to endogenously released CRF enables this population to more potently oppose CRF2R agonist activation. To reduce the total number of microinjections only a single dose of CRF1R and CRF2R agonists/antagonists were used in this work, preventing the performance of a full dose-response curve to better probe differences in male and female receptor sensitivity; this is critical future experiments in evaluating potential sex-mediate differences. Further, comparison of intact vs. ovariectomized females is of great interest in further elucidating the role of mPFC CRF receptor signaling in modulating binge ethanol intake.

An unexpected finding is that CRF1R antagonist administered into the mPFC induced a reduction in sucrose intake. As CRF1R treatment did not alter open-field activity it is unlikely a drug-induced reduction in general motor behavior mediated this effect. Further, given administration of the CRF2R agonist had no impact on sucrose intake, it is likely this change is due to alterations in CRF1R signaling alone rather than a redirection of CRF to CRF2R. A caveat to this interpretation is that, as sucrose intake was evaluated following DID ethanol testing, the lack of CRF2R agonist impact on sucrose intake may potentially be due to lingering ethanol-induced alterations in CRF2R expression/function. Further work on drug naïve animals will need to be done to address this possibility, though it is of note previous work has demonstrated drug exposure to increase, rather than decrease, CRF2R mPFC expression [31,32]. Given this caveat, the observed CRF1R specific impact is somewhat surprising as there is scarce evidence for a role of CRF1R in modulating non-stress related feeding (such as animals consuming sucrose in their homecage as used in this work) [58]. It is, however, important to note the mPFC is activated by licking behavior and projects extensively to the hypothalamus and associated regions critical for mediating general feeding behavior [59,60]. CRF1R is highly expressed on both GABAergic interneurons and glutamatergic pyramidal projection cells in the mPFC and is thus poised to potently shape mPFC output and synchronize regional neural activity via direct and indirect mechanisms [45,49,61]. Further, CRF-expressing cells within the mPFC have recently been shown to receive direct afferent connection from several areas involved in reward processing, such as the VTA and DRN [62]. Alteration in CRF signaling at CRF1R within the mPFC may therefore serve a yet to be evaluated role in the general consumption of rewarding substances. That CRF1R antagonism altered sucrose intake in a distinct temporal pattern to that which this treatment reduced ethanol intake further suggests distinct mechanisms of action. The specific populations of CRF1R-expressing cells within the mPFC engaged during binge-ethanol and sucrose consumption and their potentially divergent downstream targets which may underlie our present finding is an area of important future research.

## 5. Conclusions

Overall, in this work we show intra-mPFC microinfusion of a CRF1R antagonist or CRF2R agonist is sufficient to reduce binge-ethanol intake in male and female C57BL/6J mice. We further show CRF1R antagonist action is dependent on CRF2R availability, suggesting redirection of synaptic CRF from the CRF1 to the CRF2 receptor may underlie this effect. In conclusion, this work demonstrates a role of mPFC CRF receptors in binge-ethanol consumption in non-dependent animals, contributing to the growing understanding that alterations in CRF signaling play a key role throughout the transition from excessive ethanol intake to full dependence.

## Supplementary Materials

The following are available online at https://www.mdpi.com/2076-3425/9/7/171/s1, Figure S1: CRF2R is expressed in C57BL/6J mouse mPFC, Figure S2: mPFC CRF1R antagonism impact by sex, Figure S3: mPFC CRF2R agonism impact by sex, Figure S4: mPFC CRF1R + CRF2R antagonism impact by sex.

## References

[1] Waller M., McGuire A.C., Dobson A.J. (2015). Alcohol use in the military: Associations with health and wellbeing. Subst. Abuse Treat Prev. Policy.

[2] Rutledge P.C., Bestrashniy J.R., Nelson T.F. (2016). Problematic Drinking Among Postgraduate Students: Binge Drinking, Prepartying, and Mixing Alcohol with Energy Drinks. Subst. Use Misuse.

[3] Esser M.B., Hedden S.L., Kanny D., Brewer R.D., Gfroerer J.C., Naimi T.S. (2014). Prevalence of alcohol dependence among US adult drinkers, 2009–2011. Prev. Chronic. Dis..

[4] Fan A.Z., Russell M., Stranges S., Dorn J., Trevisan M. (2008). Association of lifetime alcohol drinking trajectories with cardiometabolic risk. J. Clin. Endocrinol. Metab..

[5] Hingson R., Heeren T., Winter M., Wechsler H. (2005). Magnitude of alcohol-related mortality and morbidity among U.S. college students ages 18–24: Changes from 1998 to 2001. Annu. Rev. Public Health.

[6] Miller J.W., Naimi T.S., Brewer R.D., Jones S.E. (2007). Binge drinking and associated health risk behaviors among high school students. Pediatrics.

[7] Thiele T.E., Crabbe J.C., Boehm S.L. (2014). “Drinking in the Dark” (DID): A simple mouse model of binge-like alcohol intake. Curr. Protoc. Neurosci..

[8] Rhodes J.S., Ford M.M., Yu C.H., Brown L.L., Finn D.A., Garland T., Crabbe J.C. (2007). Mouse inbred strain differences in ethanol drinking to intoxication. Genes Brain Behav..

[9] Zorrilla E.P., Logrip M.L., Koob G.F. (2014). Corticotropin releasing factor: A key role in the neurobiology of addiction. Front. Neuroendocrinol..

[10] Vale W., Spiess J., Rivier C., Rivier J. (1981). Characterization of a 41-residue ovine hypothalamic peptide that stimulates secretion of corticotropin and beta-endorphin. Science.

[11] Van Pett K., Viau V., Bittencourt J.C., Chan R.K., Li H.Y., Arias C., Prins G.S., Perrin M., Vale W., Sawchenko P.E. (2000). Distribution of mRNAs encoding CRF receptors in brain and pituitary of rat and mouse. J. Comp. Neurol..

[12] Slater P.G., Yarur H.E., Gysling K. (2016). Corticotropin-Releasing Factor Receptors and Their Interacting Proteins: Functional Consequences. Mol. Pharmacol..

[13] Lowery E.G., Spanos M., Navarro M., Lyons A.M., Hodge C.W., Thiele T.E. (2010). CRF-1 antagonist and CRF-2 agonist decrease binge-like ethanol drinking in C57BL/6J mice independent of the HPA axis. Neuropsychopharmacology.

[14] Lowery E.G., Thiele T.E. (2010). Pre-clinical evidence that corticotropin-releasing factor (CRF) receptor antagonists are promising targets for pharmacological treatment of alcoholism. CNS Neurol. Disord. Drug Targets.

[15] Lowery-Gionta E.G., Navarro M., Li C., Pleil K.E., Rinker J.A., Cox B.R., Sprow G.M., Kash T.L., Thiele T.E. (2012). Corticotropin releasing factor signaling in the central amygdala is recruited during binge-like ethanol consumption in C57BL/6J mice. J. Neurosci..

[16] Rinker J.A., Marshall S.A., Mazzone C.M., Lowery-Gionta E.G., Gulati V., Pleil K.E., Kash T.L., Navarro M., Thiele T.E. (2016). Extended Amygdala to Ventral Tegmental Area Corticotropin-Releasing Factor Circuit Controls Binge Ethanol Intake. Biol. Psychiatry.

[17] Koob G.F. (2013). Theoretical frameworks and mechanistic aspects of alcohol addiction: Alcohol addiction as a reward deficit disorder. Curr. Top Behav. Neurosci..

[18] Gilpin N.W., Herman M.A., Roberto M. (2015). The central amygdala as an integrative hub for anxiety and alcohol use disorders. Biol. Psychiatry.

[19] Roberto M., Gilpin N.W., Siggins G.R. (2012). The central amygdala and alcohol: Role of γ-aminobutyric acid, glutamate, and neuropeptides. Cold Spring Harb. Perspect. Med..

[20] Ketchesin K.D., Stinnett G.S., Seasholtz A.F. (2016). Binge Drinking Decreases Corticotropin-Releasing Factor-Binding Protein Expression in the Medial Prefrontal Cortex of Mice. Alcohol Clin. Exp. Res..

[21] Gass J.T., Chandler L.J. (2013). The Plasticity of Extinction: Contribution of the Prefrontal Cortex in Treating Addiction through Inhibitory Learning. Front. Psychiatry.

[22] Abernathy K., Chandler L.J., Woodward J.J. (2010). Alcohol and the prefrontal cortex. Int. Rev. Neurobiol..

[23] Klenowski P.M. (2018). Emerging role for the medial prefrontal cortex in alcohol-seeking behaviors. Addict. Behav..

[24] Lu Y.L., Richardson H.N. (2014). Alcohol, stress hormones, and the prefrontal cortex: A proposed pathway to the dark side of addiction. Neuroscience.

[25] Kubota Y., Shigematsu N., Karube F., Sekigawa A., Kato S., Yamaguchi N., Hirai Y., Morishima M., Kawaguchi Y. (2011). Selective coexpression of multiple chemical markers defines discrete populations of neocortical GABAergic neurons. Cereb. Cortex.

[26] Kubota Y., Karube F., Nomura M., Kawaguchi Y. (2016). The Diversity of Cortical Inhibitory Synapses. Front. Neural Circuits.

[27] Hupalo S., Bryce C.A., Bangasser D.A., Berridge C.W., Valentino R.J., Floresco S.B. (2019). Corticotropin-Releasing Factor (CRF) circuit modulation of cognition and motivation. Neurosci. Biobehav. Rev..

[28] Kirby L.G., Freeman-Daniels E., Lemos J.C., Nunan J.D., Lamy C., Akanwa A., Beck S.G. (2008). Corticotropin-releasing factor increases GABA synaptic activity and induces inward current in 5-hydroxytryptamine dorsal raphe neurons. J. Neurosci..

[29] Ohata H., Shibasaki T. (2011). Microinjection of different doses of corticotropin-releasing factor into the medial prefrontal cortex produces effects opposing anxiety-related behavior in rats. J. Nippon. Med. Sch..

[30] Lovenberg T.W., Liaw C.W., Grigoriadis D.E., Clevenger W., Chalmers D.T., De Souza E.B., Oltersdorf T. (1995). Cloning and characterization of a functionally distinct corticotropin-releasing factor receptor subtype from rat brain. Proc. Natl. Acad. Sci. USA.

[31] Carboni L., Romoli B., Bate S.T., Romualdi P., Zoli M. (2018). Increased expression of CRF and CRF-receptors in dorsal striatum, hippocampus, and prefrontal cortex after the development of nicotine sensitization in rats. Drug Alcohol Depend..

[32] Guan X., Wan R., Zhu C., Li S. (2014). Corticotropin-releasing factor receptor type-2 is involved in the cocaine-primed reinstatement of cocaine conditioned place preference in rats. Behav. Brain Res..

[33] Robinson S.L., Marrero I.M., Perez-Heydrich C.A., Sepulveda-Orengo M.T., Reissner K.J., Thiele T.E. (2019). Medial prefrontal cortex neuropeptide Y modulates binge-like ethanol consumption in C57BL/6J mice. Neuropsychopharmacology.

[34] Marshall S.A., McKnight K.H., Blose A.K., Lysle D.T., Thiele T.E. (2016). Modulation of Binge-like Ethanol Consumption by IL-10 Signaling in the Basolateral Amygdala. J. Neuroimmune Pharmacol..

[35] Pleil K.E., Rinker J.A., Lowery-Gionta E.G., Mazzone C.M., McCall N.M., Kendra A.M., Olson D.P., Lowell B.B., Grant K.A., Thiele T.E. (2015). NPY signaling inhibits extended amygdala CRF neurons to suppress binge alcohol drinking. Nat. Neurosci..

[36] Cox B.R., Olney J.J., Lowery-Gionta E.G., Sprow G.M., Rinker J.A., Navarro M., Kash T.L., Thiele T.E. (2013). Repeated cycles of binge-like ethanol (EtOH)-drinking in male C57BL/6J mice augments subsequent voluntary EtOH intake but not other dependence-like phenotypes. Alcohol Clin. Exp. Res..

[37] Sparrow A.M., Lowery-Gionta E.G., Pleil K.E., Li C., Sprow G.M., Cox B.R., Rinker J.A., Jijon A.M., Peňa J., Navarro M. (2012). Central neuropeptide Y modulates binge-like ethanol drinking in C57BL/6J mice via Y1 and Y2 receptors. Neuropsychopharmacology.

[38] Gilpin N.W. (2012). Neuropeptide Y (NPY) in the extended amygdala is recruited during the transition to alcohol dependence. Neuropeptides.

[39] Hwa L.S., Nathanson A.J., Shimamoto A., Tayeh J.K., Wilens A.R., Holly E.N., Newman E.L., DeBold J.F., Miczek K.A. (2015). Aggression and increased glutamate in the mPFC during withdrawal from intermittent alcohol in outbred mice. Psychopharmacology (Berl).

[40] Moorman D.E., James M.H., McGlinchey E.M., Aston-Jones G. (2015). Differential roles of medial prefrontal subregions in the regulation of drug seeking. Brain Res..

[41] Holmes A., Fitzgerald P.J., MacPherson K.P., DeBrouse L., Colacicco G., Flynn S.M., Masneuf S., Pleil K.E., Li C., Marcinkiewcz C.A. (2012). Chronic alcohol remodels prefrontal neurons and disrupts NMDAR-mediated fear extinction encoding. Nat. Neurosci..

[42] Koob G.F. (2013). Addiction is a Reward Deficit and Stress Surfeit Disorder. Front. Psychiatry.

[43] Koob G.F. (2013). Negative reinforcement in drug addiction: The darkness within. Curr. Opin. Neurobiol..

[44] Schreiber A.L., Lu Y.L., Baynes B.B., Richardson H.N., Gilpin N.W. (2017). Corticotropin-releasing factor in ventromedial prefrontal cortex mediates avoidance of a traumatic stress-paired context. Neuropharmacology.

[45] Uribe-Mariño A., Gassen N.C., Wiesbeck M.F., Balsevich G., Santarelli S., Solfrank B., Dournes C., Fries G.R., Masana M., Labermeier C. (2016). Prefrontal Cortex Corticotropin-Releasing Factor Receptor 1 Conveys Acute Stress-Induced Executive Dysfunction. Biol. Psychiatry.

[46] Hupalo S., Berridge C.W. (2016). Working Memory Impairing Actions of Corticotropin-Releasing Factor (CRF) Neurotransmission in the Prefrontal Cortex. Neuropsychopharmacology.

[47] Miguel T.T., Gomes K.S., Nunes-de-Souza R.L. (2014). Tonic modulation of anxiety-like behavior by corticotropin-releasing factor (CRF) type 1 receptor (CRF1) within the medial prefrontal cortex (mPFC) in male mice: Role of protein kinase A (PKA). Horm. Behav..

[48] Delli Pizzi S., Chiacchiaretta P., Mantini D., Bubbico G., Ferretti A., Edden R.A., Di Giulio C., Onofrj M., Bonanni L. (2017). Functional and neurochemical interactions within the amygdala-medial prefrontal cortex circuit and their relevance to emotional processing. Brain Struct. Funct..

[49] Ghosal S., Hare B., Duman R.S. (2017). Prefrontal Cortex GABAergic Deficits and Circuit Dysfunction in the Pathophysiology and Treatment of Chronic Stress and Depression. Curr. Opin. Behav. Sci..

[50] Jaferi A., Bhatnagar S. (2007). Corticotropin-releasing hormone receptors in the medial prefrontal cortex regulate hypothalamic-pituitary-adrenal activity and anxiety-related behavior regardless of prior stress experience. Brain Res..

[51] Funk C.K., Koob G.F. (2007). A CRF(2) agonist administered into the central nucleus of the amygdala decreases ethanol self-administration in ethanol-dependent rats. Brain Res..

[52] Quadros I.M., Macedo G.C., Domingues L.P., Favoretto C.A. (2016). An Update on CRF Mechanisms Underlying Alcohol Use Disorders and Dependence. Front. Endocrinol. (Lausanne).

[53] Crawley J.N., Olschowka J.A., Diz D.I., Jacobowitz D.M. (1985). Behavioral investigation of the coexistence of substance P, corticotropin releasing factor, and acetylcholinesterase in lateral dorsal tegmental neurons projecting to the medial frontal cortex of the rat. Peptides.

[54] Itoga C.A., Chen Y., Fateri C., Echeverry P.A., Lai J.M., Delgado J., Badhon S., Short A., Baram T.Z., Xu X. (2019). New viral-genetic mapping uncovers an enrichment of corticotropin-releasing hormone-expressing neuronal inputs to the nucleus accumbens from stress-related brain regions. J. Comp. Neurol..

[55] Lebow M.A., Chen A. (2016). Overshadowed by the amygdala: The bed nucleus of the stria terminalis emerges as key to psychiatric disorders. Mol. Psychiatry.

[56] Valentino R.J., Van Bockstaele E., Bangasser D. (2013). Sex-specific cell signaling: The corticotropin-releasing factor receptor model. Trends Pharmacol. Sci..

[57] Bangasser D.A., Curtis A., Reyes B.A., Bethea T.T., Parastatidis I., Ischiropoulos H., Van Bockstaele E.J., Valentino R.J. (2010). Sex differences in corticotropin-releasing factor receptor signaling and trafficking: Potential role in female vulnerability to stress-related psychopathology. Mol. Psychiatry.

[58] Stengel A., Taché Y. (2014). CRF and urocortin peptides as modulators of energy balance and feeding behavior during stress. Front. Neurosci..

[59] Horst N.K., Laubach M. (2013). Reward-related activity in the medial prefrontal cortex is driven by consumption. Front. Neurosci..

[60] Reppucci C.J., Petrovich G.D. (2016). Organization of connections between the amygdala, medial prefrontal cortex, and lateral hypothalamus: A single and double retrograde tracing study in rats. Brain Struct. Funct..

[61] Delli Pizzi S., Chiacchiaretta P., Mantini D., Bubbico G., Edden R.A., Onofrj M., Ferretti A., Bonanni L. (2017). GABA content within medial prefrontal cortex predicts the variability of fronto-limbic effective connectivity. Brain Struct. Funct..

[62] Zhang S., Lv F., Yuan Y., Fan C., Li J., Sun W., Hu J. (2019). Whole-Brain Mapping of Monosynaptic Afferent Inputs to Cortical CRH Neurons. Front. Neurosci..

