# Supplementary material for: Corticotropin Releasing Factor Type 1 and 2 Receptor Signaling in the Medial Prefrontal Cortex Modulates Binge-Like Ethanol Consumption in C57BL/6J Mice
Source: Brain Sci. 2019 Jul 19;9(7):171. doi: 10.3390/brainsci9070171 (PMC6680756; doi:10.3390/brainsci9070171)
Supplement: Supplementary file 1 [file brainsci-09-00171-s001.pdf]

METHODS

Western blots were perform as previously described [1]. In brief, four mice ≥8 weeks old (2 male and 2 female) were rapidly decapitated and brains extracted and flash frozen. A cryostat was used to collect bilateral punches of tissue (1mm x 1mm cylindrical punch) from the mPFC. Tissue punches were manually homogenized by pipetting with 100 µl lysis buffer [320 mM sucrose, 1% SDS, 5 mM HEPES buffer, and 1% Halt 100x Protease/Phosphatase Inhibitor Cocktail (Thermo Fisher Scientific, Rockford, IL)]. Protein concentrations were determined for each sample using the BCA Protein Assay Kit (Thermo Fisher Scientific), so that a total of 25 µg of total protein was loaded onto 4–20% Mini-Protean® TGX ™ Precast Protein Gels (Bio-Rad, Hercules, CA). Samples were then transferred to PVDF membranes and dried overnight. After reactivation, samples were blocked in Odyssey® Blocking Buffer (Li-COR, Lincoln, NE) for 1 h were incubated before a 2.5 h incubation with primary antibodies (1:2000 GAPDH (#2118 Cell Signaling Technology Boston, MA) and 1:2500 CRF2R (ab104368 Abcam, Cambridge, MA) in 0.2% Tween in blocking buffer at RT. Membranes were serially washed with phospho-buffered saline with 0.1% Tween-20 (PBST) before an hour incubation of anti-rabbit secondary (1:5000 secondary; 0.1%SDS, and 0.2% Tween). After a series of washes in PBST, membrane blots were imaged using Image Studio™ installed on a Dell Optiplex 7010 workstation coupled with Odyssey® FC (Li-COR). As westerns were performed for qualitative rather than quantitative analysis, images were not analyzed. Antibodies were not validated with the use of CRF2R knockout tissue.

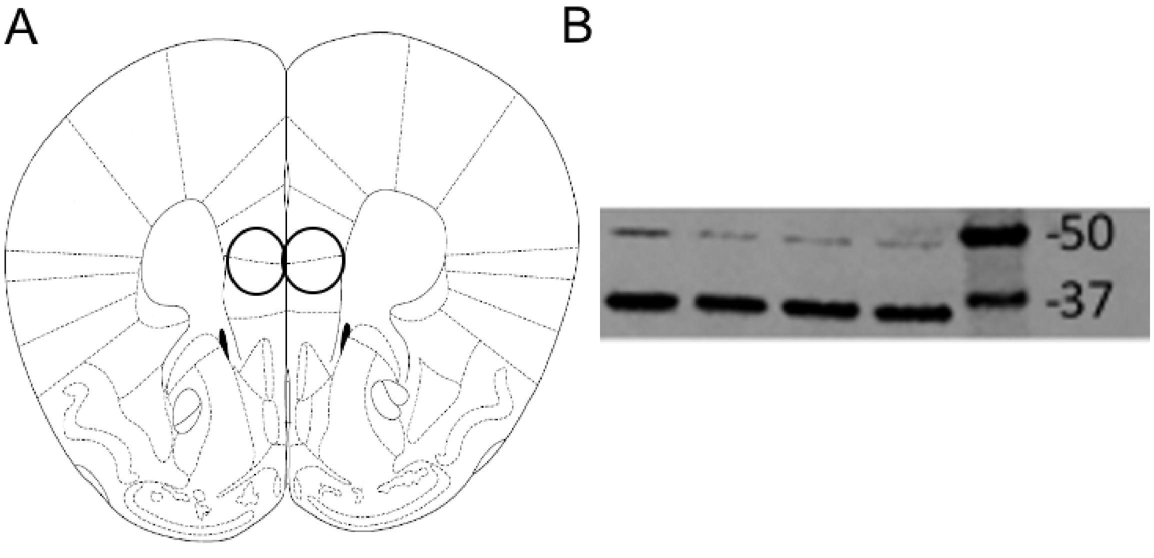

Figure S1. CRF2R is expressed in C57BL/6J mouse mPFC. **A**. Approximate area of tissue punches taken for western blot analysis. **B**. Qualitative western blot demonstrating the expression of the CRF 2 receptor in the mPFC. CRF2R band ~50; GAPDH housekeeping band ~37. Blot contains 2 male (lane 1 and 2) and 2 female (lane 3 and 4) mice.

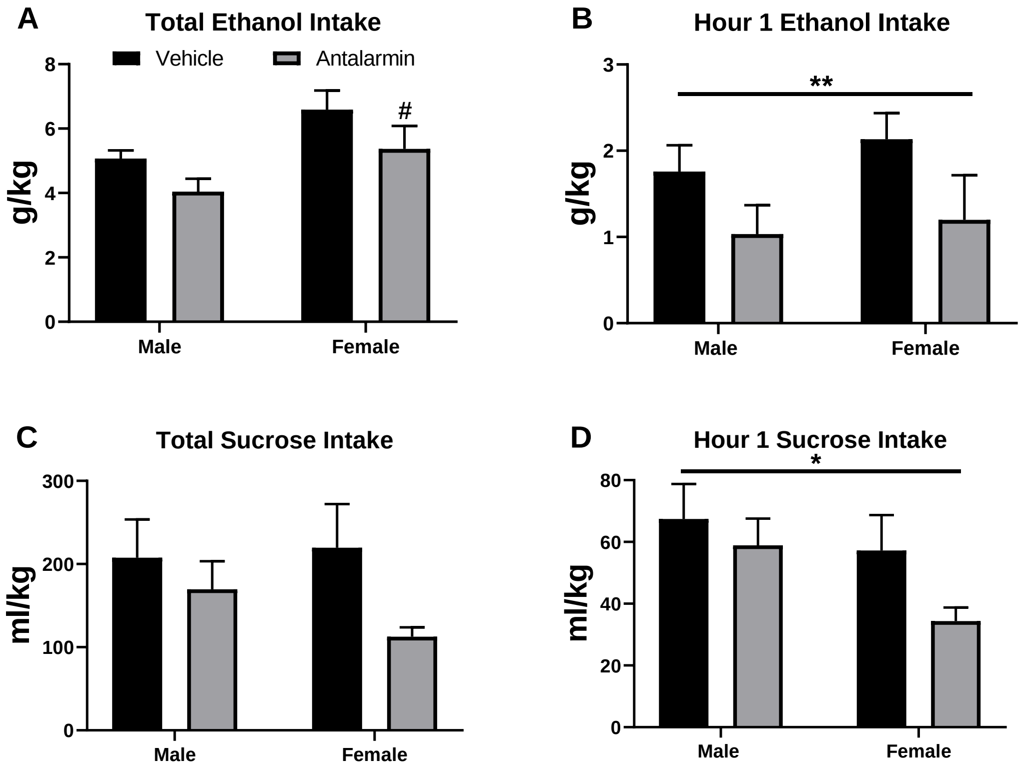

Figure S2. mPFC CRF1R antagonism impact by sex. **A**. In total ethanol intake, two-way ANOVA found a significant impact of treatment (F (1, 11) = 10.83; p < 0.01) and a trending towards significance impact of sex (F (1, 11) = 4.113; p = .07), but not an interaction between the two factors (F (1, 11) = 0.08231; p > 0.05). Given the trend towards an effect of sex, we performed Bonferroni's multiple comparisons test. This post hoc detected a sex-specific significant treatment impact in which total intake was significantly decreased in females (p < 0.05) but not males (p > 0.05). Male N = 6, Female N = 7. **B.** In the first hour of ethanol intake (when treatment had the strongest impact), two-way ANOVA found a significant impact of treatment (F (1, 11) = 10.52; p < 0. 01), but not sex (F (1, 11) = 0.3097; p > 0.05) nor an interaction between the two factors (F (1, 11) = 0.1678; p > 0.05). Male N = 6, Female N = 7. **C.** In total sucrose intake, two-way ANOVA found a trending significant impact of treatment (F (1, 10) = 4.148; p = 0.07), but not sex (F (1, 10) = 0.2595; p > 0.05) nor an interaction between the two factors (F (1, 10) = 0.9367; p > 0.05). Male N = 5, Female N = 7. **D.** In the first hour of sucrose intake, two-way ANOVA found a significant impact of treatment (F (1, 10) = 6.065; p < 0.05), but not sex (F (1, 10) = 2.197; p > 0.05) nor an interaction between the two factors (F (1, 10) = 1.272; p > 0.05). Male N = 5, Female N = 7. Two-way ANOVA treatment factor significance * = p < 0.05; ** = p < 0.01; Bonferroni's multiple comparisons test significance # = p < 0.05.

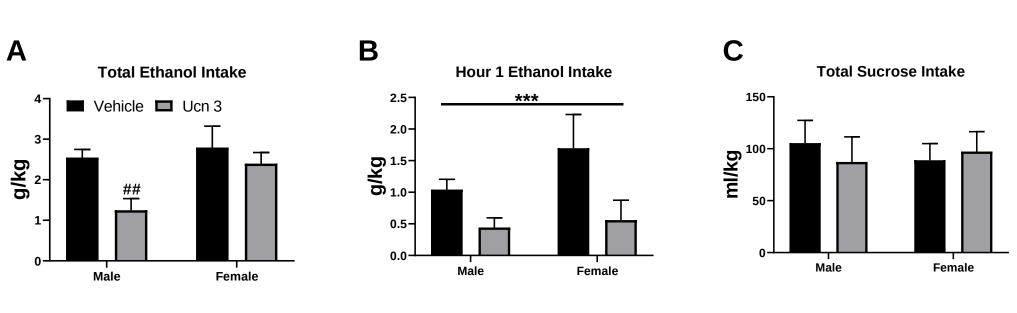

Figure S3. mPFC CRF2R agonism impact by sex. **A.** In total ethanol intake, two-way ANOVA found a significant impact of treatment F (1, 12) = 8.623; p < 0.05), but not sex (F (1, 12) = 3.994; p > 0.05) nor an interaction between the two factors (F (1, 12) = 2.432; p > 0.05). As the sex factor boarded on significance (p = 0.07), Bonferroni's multiple comparisons test was performed. Post hoc detected a sex-

specific significant treatment impact in which male ($p < 0.01$), but no female ($p > 0.05$) was significantly reduced by treatment. Male N = 9, Female N = 5. **B.** In the first hour of ethanol intake (when treatment had the strongest impact), two-way ANOVA found a significant impact of treatment ($F (1, 12) = 19.92$; $p < 0.001$), but not sex ($F (1, 12) = 1.374$; $p > 0.05$) nor an interaction between the two factors ($F (1, 12) = 1.915$; $p > 0.05$). Male N = 9, Female N = 5. **C.** In total sucrose intake, two-way ANOVA found no significant impact of treatment ($F (1, 7) = 0.1388$; $p > 0.05$), sex ($F (1, 7) = 0.01399$; $p > 0.05$) nor an interaction between the two factors ($F (1, 7) = 1.019$; $p > 0.05$). Male N = 5, Female N = 4. Two-way ANOVA treatment factor significance *** = $p < 0.001$. Sidak's multiple comparisons significance ## = $p < .001$.

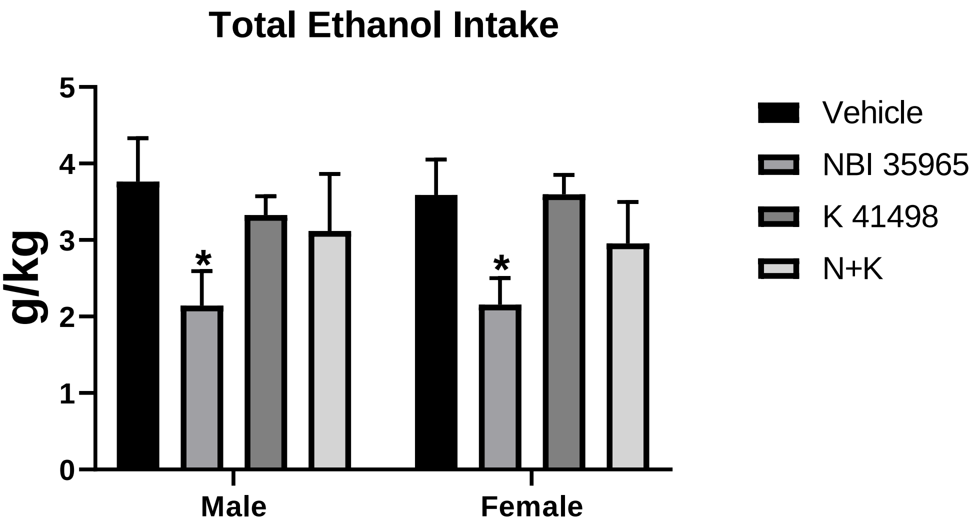

Figure S4. mPFC CRF1R+CRF2R antagonism impact by sex. **A.** In total ethanol intake, two-way ANOVA found a significant impact of treatment ($F (3, 17) = 3.486$; $p < 0.05$), but not sex ($F (1, 17) = 0.001347$; $p > 0.05$) nor an interaction between the two factors ($F (3, 17) = 0.08616$; $p > 0.05$). Vehicle male N = 3, female N = 3; NBI 35965 male N = 4, female N = 2; K 41498 male N = 5, female = 2; NBI 35965 + K 41498 male N = 4, female = 3. Two-way ANOVA treatment factor significance * = $p < 0.05$.

REFERENCES

1. Rinker, J.A.; Marshall, S.A.; Mazzone, C.M.; Lowery-Gionta, E.G.; Gulati, V.; Pleil, K.E.; Kash, T.L.; Navarro, M.; Thiele, T.E. Extended Amygdala to Ventral Tegmental Area Corticotropin-Releasing Factor Circuit Controls Binge Ethanol Intake. *Biol. Psychiatry* **2017**, *81*, 930–940.
